# Etiology and severity of various forms of ocular war injuries in patients presenting at an Army Hospital in Pakistan

**Published:** 2017

**Authors:** Syed Abid Hassan Naqvi

**Affiliations:** Dr. Syed Abid Hassan Naqvi, FCPS. Department of Ophthalmology, Armed Forces Institute of Ophthalmology, Peshawar, Pakistan

Ocular trauma is an evolving sub-specialty. Over the last two decades, various societies and groups of consultants have devised standardized and unambiguous terminologies, classifications and predictive models to keep all ophthalmologists on the same grid.^1^,^2^ Naqvi et al in their above mentioned manuscript published in Pakistan Journal of Medical Sciences Vol. 32 (6) 2016, have highlighted the etiology and severity of various forms of ocular war injuries. Ocular war injuries are bilateral in 15-25% of cases.^3^ In a study, where IED blast and splinter injuries are the most frequent mode of injury; not a single case of bilateral ocular injury is quite unusual and in contradiction to published data. Moreover, Birmingham Eye Trauma Terminology (BETTS) is the standardized and acceptable classification of mechanical eye injuries which is not followed in the study. As per BETTS, the word ‘blunt’ should be replaced by one of the more appropriate terms i.e ‘contusion’ or ‘rupture’. Otherwise the ambiguity remains whether the inflicting object is blunt or the consequences of trauma are blunt.

According to BETTS, the term ‘rupture’ is an open globe injury due to blunt trauma. In Table-I none of the patient had open globe injury due to blunt trauma, while in Table-III, it is mentioned that six patient had rupture, which is not understandable. Ocular trauma score (OTS) is a predictive model that can predict final visual outcome on the basis of initial raw score recorded at the time of injury. On the basis of raw score patient is divided into one of the 5 different categories (1-5), rather than grades (I -V). Moreover, OTS has been widely used as a predictive model in open globe injuries. Head to head comparison of OTS in open versus closed globe injuries is not a valid option. However, OTS can be used to validate the final visual outcome separately in open and closed globe injuries.

***Surg Cdr Qamar Ul Islam Eye Specialist PNS Shifa, Karachi qamarulislam71@gmail.com***

## Response from the authors

We believe that healthy criticism is the ladder to developing best medical experiences which would benefit the patient. We are thankful to the critique for taking special interest in analyzing our article. Regarding the points raised, I would like to humbly submit that the critique has very rightly said that 15 – 25% cases of war ocular injuries are bilateral. In fact, we did receive information about bilateral ocular injuries but they had been evacuated to other triage facilities in the vicinity owing to other serious chest/abdominal injuries. A number of patients had bilateral ocular injury but they had received trauma in one eye with peri-ocular/adnexal injuries in the other eye. Since adnexal injuries are not covered by OTS, these patients were thus labeled as unilateral ocular injury. Moreover the cases of globe rupture that we did encounter were those that occurred secondarily due to the pressure wave effect of the blasts mostly with no obvious penetrating inciting injury.

Birmingham Eye Trauma Terminology System (BETTS) was devised by the Ocular Trauma Classification Group. As a next step, the group studied 100 variables of 2500 ocular injuries in Hungary and United States of America. After such extensive studies, Ocular Trauma Score (OTS) was developed by Kuhn et al.^1^ OTS has widely been accepted as a standard for depicting prognosis six months after injury. Since after every ocular injury, the main concern of the patient and their relatives as well as ophthalmologists is to predict the visual prognosis, OTS was used in our study. Moreover, while describing OTS, Kuhn F et al. have used the terminology “serious eye injury” which covers both the open globe and close globe injuries.

The term grade has been derived to describe the OTS categories. Since, the grades of OTS in this study were corresponding to OTS categories, hence the term grade was used. In fact, a researcher even used the term OTS class to describe the OTS category.^2^ Though mostly roman numerals have not be used that often to denote the grades or categories.

Ocular trauma is a vast universe in itself. Victims of blast injuries do undergo post-traumatic stress disorder which can be worsened by poor vision. In this regard, OTS has helped us in managing trauma victims by predicting the prognosis of ocular injury. Even then the OTS has its limitations as pointed out earlier as it does not cater for the chemical injuries, thermal injuries and adnexal injuries but so fat that’s the best predictive tool we have and in a war setup it’s the most practical scoring system for us.
